# The anti-cancer role of microRNA-143 in papillary thyroid carcinoma by targeting high mobility group AT-hook 2

**DOI:** 10.1080/21655979.2022.2044277

**Published:** 2022-02-25

**Authors:** Chao Ding, Tiefeng Shi, Gang Wu, Jianting Man, Hongyu Han, Yunfu Cui

**Affiliations:** Departments of General Surgery, The Second Affiliated Hospital of Harbin Medical University, Harbin, Heilongjiang, China

**Keywords:** Papillary thyroid carcinoma (PTC), miR-143, HMGA2, cancer progression

## Abstract

Papillary thyroid carcinoma (PTC), a common thyroid cancer (TC) subtype, rapidly increases in occurrence. MicroRNAs (miRNAs), which are non-coding small RNAs, have been demonstrated to play a role in cancer pathogenic mechanisms. Although miR-143 is involved in suppressing certain malignant tumor progression, its biological role is unknown in PTC. The present study found that miR-143 levels were strongly lower in PTC patient samples and cell lines, implying that miR-143 may play a biological role in PTC. Down-regulation of miR-143 resulted in the increased expression of HMGA2. Furthermore, HMGA2 was found to be a direct target of miR-143. A dual-luciferase assay confirmed a direct binding site for miR-143 was confirmed on HMGA2 using a dual-luciferase assay. Next, over-expression of miR-143 suppressed PTC cell growth as analyzed by MTT, clone formation, and Ki-67 immunofluorescence staining assays. miR-143 mimics transfection downregulated the expression of PCNA, CDK4, CDK1, and Cyclin E1. In addition, wound healing and trans-well assays revealed that miR-143 up-regulation inhibited PTC cells invasion and migration. Co-transfection of HMGA2 expression vector restored HMGA2 expression and rescued PTC cells proliferation capability in miR-143 mimics transfected PTC cells, indicating that miR-143 inhibited PTC cells proliferation via HMGA2. These observations were also obtained in xenografts experiments in nude mice. Altogether, our study shed light on miR-143ʹs anti-cancer biological functions in PTC progression through targeting HMGA2, suggesting that restoration of miR-143 could be a potential therapeutic approach for PTC treatment.

## Graphical abstract

**Figure:**
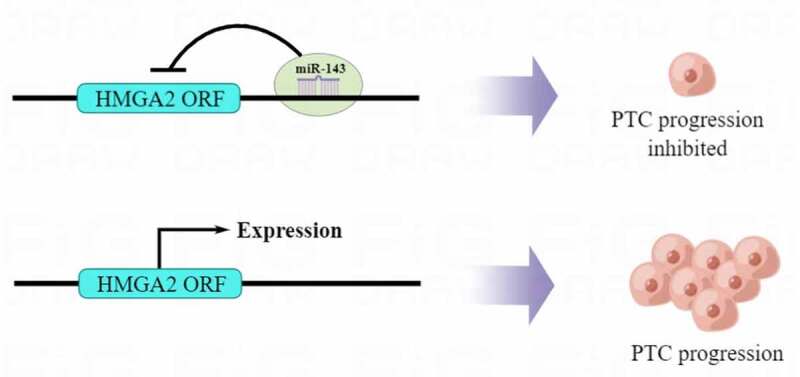

## Introduction

Thyroid cancer is a common endocrine cancer that affects people worldwide [^1–3^]. The most prevalent type of thyroid cancer is papillary thyroid carcinoma, accounting for 90% of all thyroid cancer cases (PTC) [^4–6^]. A previous study showed that the global mortality of thyroid cancer has remained relatively consistent since the 1970s. However, its incidence has been rapidly growing, which is mainly due to the increasing incidence of PTC. Although approximately 90% of PTC cases have good long-term survival, 20% of patients will relapse [7]. Even though many studies have identified that certain genes participate in PTC development, the pathogenic mechanism must be illustrated further [8].

microRNAs (miRNAs or miRs) is a class of non-coding RNAs with length of 16–24 nucleotides. MiRNAs regulate gene expression at post-transcriptional level by interacting with related mRNAs, causing degradation and suppression [^9–11^]. Several studies have demonstrated that miRNAs, such as miR-let-7e, miR-320, and miR-151, play important biological roles in the clinicopathological characteristics of PTC. MiR-143 is demonstrated to play a tumor suppressor role in multiple cancers [^12–14^]. Deep sequencing technologies have shown that miR-143 is one of the reduced miRNAs in triple-negative breast cancer [15]. MiR-143 has been found to suppress colorectal cancer cells invasion and migration by targeting MACC1 [16]. Inhibition of miR-143 promotes prostate cancer progression [17]. Growing evidence suggests that miR-143 is down-regulated in PTC. However, just one study reported that targeting MSI2 with upregulation of miR-143 induces PTC cells to apoptosis and suppresses PTC progression [18]. Thus, further investigations and more evidences are needed to explore the precise molecular mechanism of miR-143 in PTC.

High mobility group AT-hook 2 (HMGA2) is an important ubiquitously expressed transcription modulator with a higher expression in the thyroid. HMGA2 functions as a chromatin architectural factor and plays a relevant and causal role in cancer onset and development by virtually influencing all cancer hallmarks [19]. PTC has a higher level of HMGA2 mRNA [19], and HMGA2 expression in thyroid nodules could be a marker for thyroid cancer preoperative diagnosis [20]. Some researchers have reported that miR-204 and let-7 can limit PTC progression by reducing HMGA2 expression [21,22]. However, more research is required into the mechanism of HMGA2 up-regulation in PTC.

Our bioinformatics analysis revealed HMGA2, an important transcription regulator with pro-oncogenic functions in various cancers. In the present study, we aimed to explore the anti-cancer role and related molecular mechanism of miR-143 in PTC. We hypothesized that miR-143 inhibited PTC progression by targeting HGMA2. Our data revealed that miR-143 levels are decreased in PTC patients, consistent with previous studies. Furthermore, over-expression of miR-143 suppressed PTC cell proliferation, cell invasion, and metastasis. HMGA2 is the direct target of miR-143ʹs, and its over-expression down-regulated HMGA2. Exogenous HMGA2 expression restored proliferation capability in miR-143 over-expressed PTC cells. These results improve our understanding of miR-143 anti-cancer function and the underlying mechanism for PTC progression by inhibiting HMGA2 and suggest a potential PTC therapeutic target.

## Materials and methods

### PTC tissues

Between July 2018 and February 2019, the surgically resected PTC and matched non-carcinoma specimens were collected from 15 PTC cases at the Second Affiliated Hospital of Harbin Medical University (Harbin, China). The current study’s procedure was approved by Harbin Medical University’s Ethics Committee (HMUIRB20150023). These patients ranged in age from 40 to 77 years old (on average, 43.4). Each patient gave informed consent for tissue collection. Each tissue sample was instantly frozen and stored in liquid nitrogen before being used in this research.

### Cell culture

We cultured human thyroid epithelial Nthy-ori3-1 cells and PTC TPC-1 cells within RPMI-1640 medium (GIBCO, USA) that contained 10% fetal bovine serum (FBS; GIBCO, USA) as well as 1% penicillin G/streptomycin (GIBCO Laboratories, Grand Island, NY, USA), followed by incubation under 37°C and 5% CO_2_ conditions.

### Cell transfection

We acquired sequences of miR-143 mimics (Cat# B02003) and their corresponding control from GenePharma, Shanghai, China.

We inoculated TPC-1 cells in the 6 cm dishes at 24 h before transfection. Lipofectamine 2000 (Invitrogen, USA) was adopted in 5 h transfection of cells using miR-143 mimics or mimic-NC with the serum-free medium in line with specific instructions. Then, the original medium was replaced by the complete one. We collected cell lysates at 48 h post-transfection.

### Quantitative real-time PCR analysis (qRT-PCR)

We extracted total RNA using TRIzol reagent (Invitrogen, Carlsbad, CA) according to the manufacturer’s instructions. After that, total RNA (1 g) was reverse transcribed into cDNA with the help of Superscriptase II (Invitrogen, USA). We then used Power SYBR Green PCR Master Mix to perform qRT-PCR (Life Technologies; Thermo Fisher Scientific, Waltham, MA, USA).

The endogenous reference was U6 small nuclear (sn)RNA, and the miR-143 level was determined using a 2^-ΔΔCT method. miR-143 (F: 5’- GGGTGAGATGAAGCACTGTAGCTC-3’; R: 5’- GCTGTCAACATACGCTACGTAACG −3’), human U6 (F: 5’- CGCTTCACGAATTTGCGTGTCA −3’; R: 5’- GCTTCGGCAGCACATATACTAAAAT −3’). U6 snRNA expression was applied in normalizing gene expression.

### Western blotting (WB) assay

The protein levels were examined using a Western blotting assay [23]. Cell lysis was conducted using the protease inhibitor cocktail-containing RIPA lysis buffer (Roche, Switzerland). Bradford’s approach was utilized to measure protein content. Equivalent protein volumes were isolated through SDS-PAGE and then transferred onto PVDF membranes (Thermo Scientific, USA). Afterward, we blocked membranes using 5% nonfat milk, anti-HMGA2 (#8179) (Cell signaling technology, USA), anti-Cyclin E1 (sc-377100), anti-PCNA (sc-56), anti-Actin (sc-8432), anti-CDK1 (sc-53217), and anti-CDK4 (sc-377100) (Santa Cruz, USA) primary antibodies were used to incubate blots. Later, blots were washed and further incubated using anti-mouse (#7076) or anti-rabbit (#7074) secondary antibodies (Cell signaling, USA), followed by visualization with ECL reagent (GE Healthcare, USA).

### Dual-luciferase reporter assay

The interaction between miR-143 and HMGA2 was analyzed by a dual-luciferase reporter assay [23]. The potential binding site of miR-143 on HMGA2 was predicted by Targetscan and microrna.org databases. Wild-type binding sites and flanking sequence (~200bp) were cloned into the pMIR-REPORT Luciferase vector at HindIII and SpeI sites (Ambion; Thermo Fisher Scientific). To generate the pMIR-REPORT Luciferase vector carrying the miR-143 binding site mutant sequence, Phusion Site-Directed Mutagenesis Kit (Thermo Fisher Scientific) was used to introduce several point mutations into the HMGA2 3’-UTR. The correct sequences of HMGA2-WT and HMGA2-Mut were confirmed by DNA sequencing.

After that, we inoculated TPC-1 cells into the 6-well plates, followed by transfection using Lipofectamine 2000 (Invitrogen) by specific vectors for 48 h. Afterward, we adopted a Dual-Luciferase-reporter 1000 detection system (Promega, Madison, WI, USA) to detect luciferase activities normalized to Renilla.

### 3-(4,5-Dimethylthiazol-2-yl)-2,5- diphenyltetrazolium bromide assay (MTT assay)

TPC-1 cells transfected with miR-143 mimics or mimics-NC were placed in each well of 96-well plates (5000 cells/well). On days 0, 2, and 4 post-transfection, 5 mg/mL MTT (20 μL) dissolved in PBS (Sigma, St Louis, MO, USA) was used to stain cells for a 4 h period under 37°C. We added 150 μL dimethyl sulfoxide (DMSO) into all wells following careful aspiration. After that, we detected an absorbance (OD) value at 490 nm.

### Colony formation assay

For investigating the ability to form colonies, we inoculated 800 transfected TPC-1 cells in the 6 cm dishes, followed by 14 days of culture. Thereafter, methanol was utilized for colony fixation, whereas 0.1% crystal violet (Sigma, St Louis, MO, USA) was utilized for staining.

### Ki-67 immunofluorescence staining

We plated TPC-1 cells onto the coverslips, followed by transfection using miR-143 mimics or corresponding NC for 48 h. Afterward, we fixed cells and added a Ki-67 antibody (Cell Signaling Technology, USA) to further incubate for a 20-min period under ambient temperature. Later, DAPI was utilized to counterstain cell nuclei. Each coverslip was sealed with a prolong® diamond antifade mountant (Applied Biosystems, USA).

### Wound-healing assay

We plated the miR-143 mimics or corresponding NC-transfected TPC-1 cells into 3.5 cm dishes and grew them to 70%-80%. Artificial wounds were created with the 200 μL pipette tip. We determined the migration distance at 48 hours after scratching.

### Trans-well assay

To carry out migration assays, we plated the miR-143 mimics or corresponding NC-transfected TPC-1 cells into the top 24-well Transwell chamber (5 × 10^4^ cells/well) containing the 8-μm polycarbonate nucleopore filters (Corning Costar, Corning, NY, USA). The top chamber was added with a serum-free medium, while the bottom had a 10% FBS-containing medium. Later, we incubated cells for 24 h under 37°C with 5% CO_2_ conditions. We then fixed cells attached to the lower filter surface and counted them with crystal violet staining.

In invasion assays, Matrigel (40 μL, BD Biosciences, USA) was coated on the Transwell chamber ahead of time, followed by 4 h incubation under 37°C for the formation of the basement membrane. The remaining analyses were identical to those in the migration assays.

### Nude mice

We obtained the 6-8-week-old athymic BALB/c nude mice in Vital River Laboratory, Beijing, China, and raised them under SPF condition.

Nude mice were given a subcutaneous injection of miR-143 mimics or corresponding NC-transfected or HMGA2 over-expression vector co-transfected TPC-1 cells (5 × 10^6^) via the right flank. The caliper was utilized to measure tumor size at 7-day intervals. We determined the lengths of tumor maximum (L) and minimum (W) to calculate tumor size by ½LW^2^. Three weeks later, we killed each animal to dissect the xenograft for later analyses.

### Statistical analysis

Results from 3 separate assays were displayed in the form of mean ± SD. We used SPSS17.0 (SPSS, Chicago, IL, USA) in statistical analyses. Two-tailed Student’s t-test was utilized to compare between groups, whereas ANOVA was employed to compare multiple groups. P < 0.05 stood for statistical significance.

## Results

In the present study, we intended to investigate the role and mechanism of miR-143 in preventing PTC progression. A series of in vitro and in vivo assays revealed that miR-143 inhibited PTC cell proliferation and metastasis by targeting HMGA2.

### MiR‐143 is decreased within PTC tissues and directly suppresses HMGA2 expression

**Figure 1.**
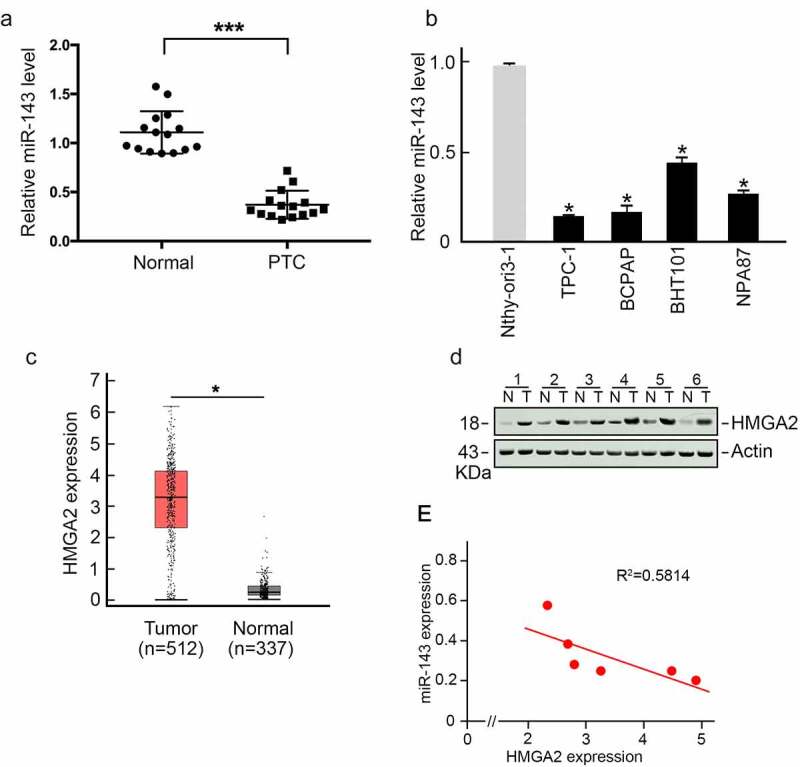
miR-143 showed a negative relation to HMGA2 level within PTC. (a) In comparison to paired healthy tissues, the expression of miR-143 was lower in PTC. *p < 0.05. (b) In comparison with healthy Nthy-ori3-1 cells, miR-143 expression declined in different PTC cells. *p < 0.05. (c) HMGA2 level markedly elevated within PTC. TCGA-derived data. *p < 0.05. (d) HMGA2 protein expression elevated within PTC. (e) miR-143 level showed negative relation to HMGA2 level.

To analyze miR-143 expression in PTC, we collected 15 PTC tissues and matched non-carcinoma samples. We conducted qRT-PCR assays to measure miR-143 levels. As displayed in Figure 1(a), miR-143 expression in PTC samples was significantly lower than in matched non-carcinoma samples. Additionally, we observed that miR-143 expression was reduced in various PTC cells compared to healthy Nthy-ori3-1 cells (Figure 1(b)). TCGA dataset analysis using Gene Expression Profiling Interactive Analysis (GEPIA) [24] revealed that HMGA2 expression, a critical oncogenic regulator, was increased in PTC samples (Figure 1(c)). HMGA2 was also remarkably increased in PTC patient samples compared to matched non-carcinoma samples (Figure 1(d)), which was negatively correlated with miR-143 expression (Figure 1(e)).

**Figure 2.**
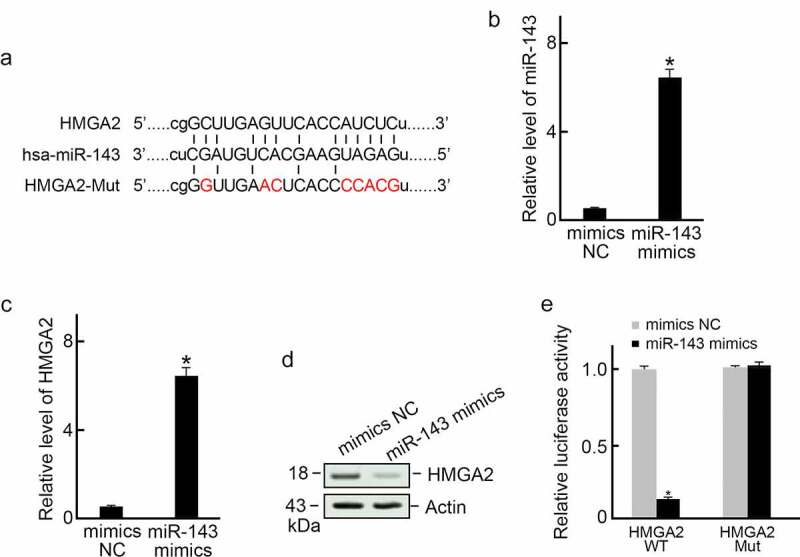
HMGA2 served as miR-143ʹs direct target. (a) Sketch map showing the possible miR-143 binding site in the HMGA2 3’-UTR. (b) miR-143 mimics transfection and increased expression of miR-143 in TPC-1 cells. *p < 0.05. (c) miR-143 overexpression decreased the HMGA2 mRNA level in TPC-1 cells. *p < 0.05. (d) miR-143 overexpression reduced the HMGA2 protein level in TPC-1 cells. (e) Dual-luciferase reporter assay. miR-143 mimic or mimics-NC was co-transfected with HMGA2-MUT or HMGA2-WT 3’-UTR of HMGA2 into TPC-1 cells. At 48 h post-transfection, we measured relative luciferase activities. n = 3. *p < 0.05.

Based on bioinformatics database analysis (Targetscan and microrna.org), we found that HMGA2 is a target candidate of miR-143 (Figure 2(a)). We then transfected TPC-1 cells using mimics-NC or miR-143 mimics sequence, respectively (Figure 2(b)). The results revealed that miR-143 over-expression decreased HMGA2 expression levels (Figure 2(c,d)). Next, we examined whether miR-143 inhibited HMGA2 expression at the predicted binding site. In the dual-luciferase reporter assay, we found that miR-143 mimics dramatically inhibited luciferase activity in HMGA2-WT group but had no effect in the predicted binding site mutant group (Figure 2(e)).

Taken together, our data suggest that miR-143 expression decreased; however, HMGA2 expression is increased in PTC, and miR-143 over-expression can suppress HMGA2 expression.

### Over-expression of miR-143 inhibits PTC cells proliferation

**Figure 3.**
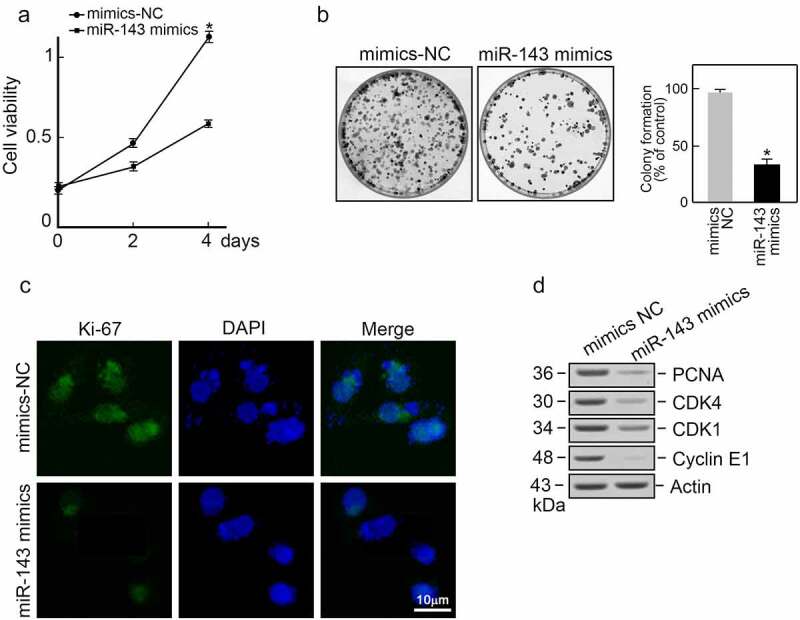
miR-143 overexpression suppresses the proliferation of PTC cells. (a) miR-143 overexpression significantly decreased the viability of TPC-1 cells. *p < 0.05. (b) miR-143 overexpression suppressed TPC-1 clone forming ability. Representative plates were shown (left panel) and the number of colonies were counted (right panel). *p < 0.05. (c) Ki-67-based immunofluorescence staining on miR-143 mimics or corresponding NC-transfected TPC-1 cells. (d) Cyclin E1, PCNA, CDK1, and CDK4 expression within transfected TPC-1 cells was analyzed by immunoblotting, with actin serving as a control.

We transfected TPC-1 cells using miR-143 mimics or mimics-NC sequence and then carried out various experiments to determine miR-143ʹs biological role in PTC. According to MTT assay, miR-143 mimics strongly inhibited PTC cells viability (Figure 3(a)). Moreover, miR-143 overexpression resulted in fewer colonies in the formation assay (Figure 3(b)). Immunofluorescence staining Ki-67, a proliferation marker, exhibited a lower signal in the miR-143 mimics group compared to the mimics-NC group (Figure 3(c)). Moreover, over-expression of miR-143 reduced protein levels of cell cycle promoting factors, including PCNA, CDK4, CDK1, and Cyclin E1 (Figure 3(d)). Based on the above results, miR-143 can inhibit PTC cells proliferation.

### miR-143 over-expression suppresses PTC cells metastasis

**Figure 4.**
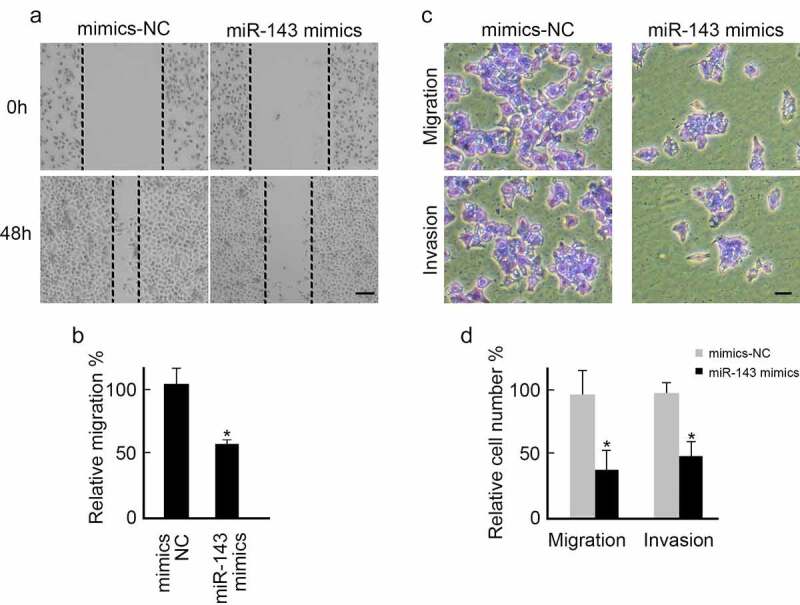
Over-expression of miR-143 inhibits PTC cells metastasis. (a) TPC-1 cells transfected with miR-143 mimics or mimics-NC were subjected to wound healing assay. Representative images were shown. scale bar = 250 μm. (b) Over-expression of miR-143 decreased wound healing speed in TPC-1 cells analyzed from (A). *p < 0.05. (c) TPC-1 cells transfected with miR-143 mimics or mimics-NC were subjected to the trans-well assay with or without Matrigel. Representative images were shown. scale bar = 100 μm (d) Over-expression of miR-143 decreased TPC-1 cells migration and invasion analyzed from (C).

We also investigated the biological role of miR-143 in regulating PTC cells metastasis. Scratch and Transwell assays were performed using miR-143 mimics or mimics-NC sequence-transfected TPC-1 cells. Over-expression of miR-143 slowed healing speed than in the mimics-NC group (Figure 4(a,b)). miR-143 overexpression also inhibited TPC-1 cells migration and invasion (Figure 4(c,d)). Taken together, miR-143 can suppress PTC cells’ metastasis.

### MiR-143 suppresses PTC cells proliferation through HMGA2

**Figure 5.**
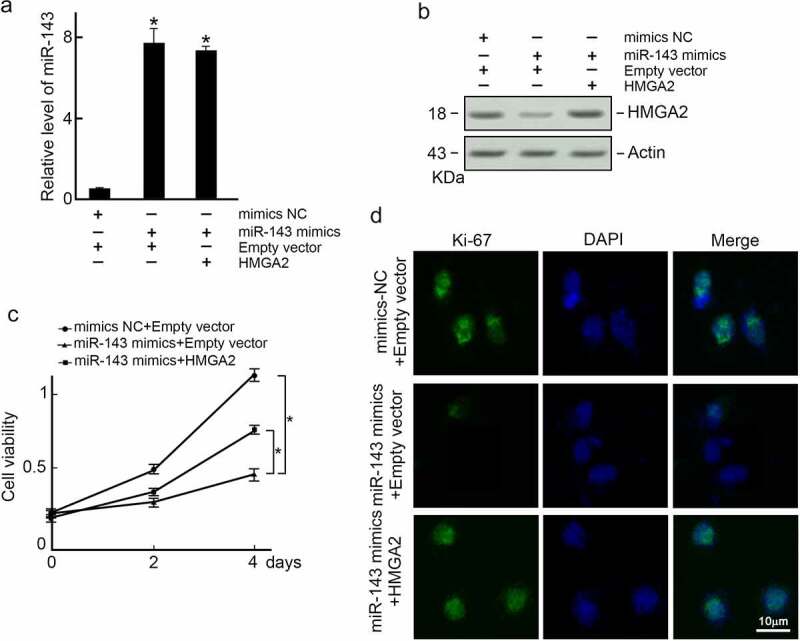
Exogenous expression of HMGA2 restores proliferation capability in miR-143 over-expressed TPC-1 cells. TPC-1 cells were co-transfected with indicated reagents, and (a) Levels of miR-143 were examined by qRT-PCR. *p < 0.05; (b) HMGA2 expression was examined by immunoblotting. Actin was served as a loading control. (c) Exogenous expression of HMGA2 restored cell growth capabilities in TPC-1 cells over-expressed with miR-143. *p < 0.05. (d) Exogenous expression of HMGA2 restored Ki-67 expression in miR-143 over-expressed TPC-1 cells.

To further explore whether miR-143 reduces PTC progression via HMGA2, TPC-1 cells were co-transfected with miR-143 mimics and HMGA2 expression vector. We found that miR-143 expression was increased by mimics transfection (Figure 5(a)), and HMGA2 protein level was decreased (Figure 4(b)). When miR-143 over-expressed TPC-1 cells were co-transfected with an HMGA2 expression vector, the level of HMGA2 was reduced (Figure 5(b)). However, restoration of HMGA2 expression reversed cell proliferation in miR-143 over-expressed TPC-1 cells as observed in the MTT assay (Figure 5(c)) and Ki-67 immunofluorescence staining (Figure 5(d)). Collectively, miR-143 can suppress PTC cells growth via HMGA2.

### MiR-143 suppresses PTC progression in vivo through HMGA2

**Figure 6.**
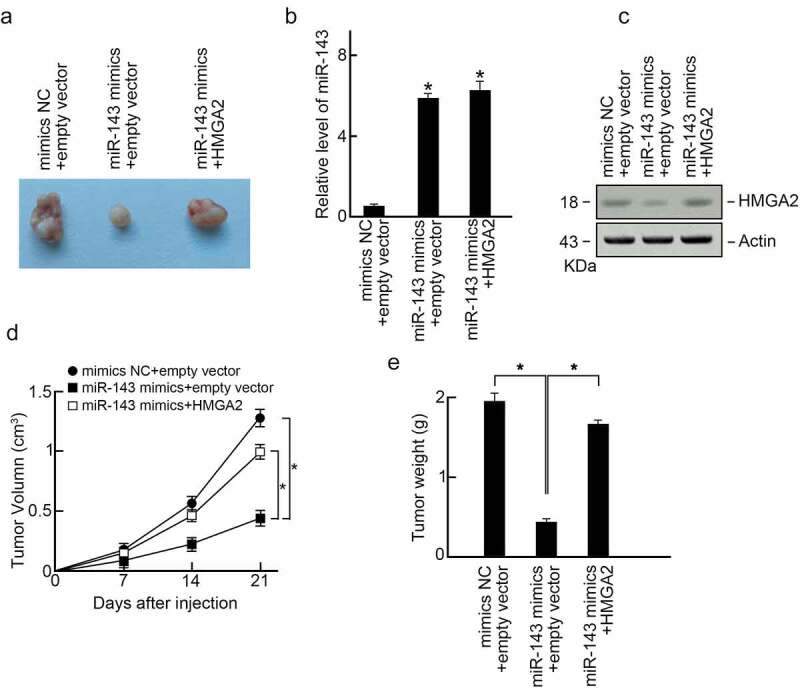
MiR-143 suppresses PTC progression in vivo through HMGA2 (a) Xenograft tumors formed in nude mice. A total of 5 × 10^6^ cells was subcutaneously injected into nude mice (n = 3). The mice were sacrificed on day 21 after the injection. Tumors were isolated, and representative images were shown. (b) Expression of miR-143 in xenograft tumors. (c) Expression of HMGA2 in xenograft tumors. (d) Volumes of xenograft tumors. (e) Weight of xenograft tumors.a.

To determine miR-143ʹs anti-cancer activity in PTC in vivo, miR-143 mimics or mimics-NC-transfected or HMGA2 expression vector co-transfected TPC-1 cells were given into nude mice via subcutaneous injection by flanks. After three weeks, we killed the animals to dissect xenografts (Figure 6(a)). In the comparison control group, miR-143 expression in xenografts elevated in miR-143 mimics groups as well as miR-143 mimics and HMGA2 co-transfection groups (Figure 6(b)). In addition, the level of HMGA2 in xenografts was reduced in miR-143 mimics groups compared to control groups, while HMGA2 expression vector co-transfection abolished HMGA2 expression within xenografts (Figure 6(c)). We found that miR-143 over-expression significantly inhibited xenograft’s size and weight compared to the control group (Figure 6(d,e)). As expected, co-over-expression of HMGA2 reversed PTC progression in the miR-143 mimics group (Figure 6(d,e)). As a result, miR-143 inhibited PTC progression in vivo through HMGA2.

## Discussion

The most frequent endocrine cancer is thyroid carcinoma, which is dominated by PTC [25,26]. The incidence of thyroid carcinoma, in particular PTC, shows an increasing tendency [27]. In the present study, we focused on miR-143, a markedly reduced miRNAs in PTC. We observed that miR-143 expression significantly decreased in PTC cells and PTC patient samples. These findings demonstrate the anti-cancer role of miR-143 in PTC.

Many studies have shown that miR-143 has an anti-cancer effect in various cancers. For instance, a recent report found that miR-143 is a tumor suppressor in human bladder cancer [28]. In renal cell cancer, miR-143 inhibits K-RAS signaling, resulting in considerable growth suppression [29]. Glioblastoma stem-like cells lose stemness when miR-143 is over-expressed [30]. Some studies reported that various non-coding RNAs promote cancer by sponging miR-143 [^31–33^]. All these studies were conducted on various cancers except PTC, and based on these studies, and we hypothesized that miR-143 might have an anti-cancer role in PTC. In the present study, miR-143 over-expression inhibited PTC cells proliferation, invasion, and migration, suggesting its tumor suppressor biological in PTC. Consistently, in vivo xenografts experiments also revealed that miR-143 over-expression inhibited PTC progression. These findings provided novel supporting evidences of anti-cancer effects of miR-143 in PTC. Another study found that miR-143 can promote cancer progression, which contradicts our findings. A high level of miR-143 enhances hepatocarcinoma metastasis via repressing fibronectin expression [34]. The different activities of miR-143 in PTC and hepatocarcinoma suggest tissue-specific or cancer-specific effects.

HMGA2 is an important member of the non-histone chromosomal high mobility group (HMG) protein family. HMGA2 modulates gene transcription by binding to AT‑rich sequences in DNA minor grooves [35]. HMGA2 has been shown to be a crucial regulator in cancers in a number of previous studies [36]. It promotes cancer cells proliferation by accelerating the cell cycle and inhibiting apoptosis [^37–39^]. Loss of HMGA2 inhibits cancer progression [40,41]. In our study, we found that HMGA2 expression strongly increased in PTC patient samples which was negatively correlated with miR-143 level in PTC patient samples. Reduced level of miR-143 resulted in great higher expression of HMGA2, while over-expression of miR-143 repressed HMGA2 expression. Bioinformatic analysis and luciferase reporter assay demonstrated that HMGA2 is a direct target of miR-143. These findings elucidated a reasonable mechanism for HMGA2 up-regulation in PTC. Moreover, exogenous expression of HMGA2 could reverse miR-143 over-expression induced growth arrest both in vitro and in vivo, implying that miR-143/HMGA2 axis is vital for PTC progression.

## Conclusion

Taken together, our recent study, which used various in vitro and in vivo assays, elucidated that targeting HMGA2 has a key anti-cancer role in PTC. Our research contributed to a better understanding of miR-143ʹs role in PTC pathogenesis. Also, we reported a novel pathway for HMGA2 up-regulation in PTC via miR-143 down-regulation.

## Data Availability

The data that support the findings of this study are available from the corresponding author upon reasonable request.
